# Developing a scale to measure family dynamics related to long-term care, and testing that scale in a multicenter cross-sectional study

**DOI:** 10.1186/1471-2296-15-134

**Published:** 2014-07-10

**Authors:** Tesshu Kusaba, Kotaro Sato, Yoshinori Matsui, Satoshi Matsuda, Takashi Ando, Ken Sakushima, Takafumi Wakita, Shingo Fukuma, Shunichi Fukuhara

**Affiliations:** 1 Hokkaido Center for Family Medicine, 1-18 Kita 41-Jo Higashi 15 Cho-me, Higashi-ku, Sapporo, Japan; 2 Department of Neurology, Hokkaido University Graduate School of Medicine, Kita-ku, Sapporo N15W7, Japan; 3 Faculty of Sociology, Kansai University, Yamate-cho, Suita-shi, Osaka 3-3-35, Japan; 4 Institute for Health Outcomes and Process Evaluation Research, Akinonocho, Nakagyo Ward, Kyoto, Japan; 5 Department of Healthcare Epidemiology, School of Public Health in the Graduate School of Medicine, Kyoto University, Yoshida Konoemachi, Sakyo-ku, Kyoto 606 8501, Japan; 6 Center for Innovative Research for Communities and Clinical Excellence (CIRC2LE), Fukushima Medical University, 1 Hikariga-oka, Fukushima City, Japan

**Keywords:** Family dynamics, Scale, Family caregivers, Long-term care, Care burden, Family medicine, Factor analysis, Criterion-related validity, Reliability, Family dynamics index for long-term care

## Abstract

**Background:**

As Japan’s population ages, more frail elderly people are cared for by members of their family. The dynamics within such families are difficult to study, in part because they are difficult to quantify. We developed a scale for assessing family dynamics related to long-term care. Here we report on the development of that scale, and we present the results of reliability testing and validation testing.

**Methods:**

Two primary-care specialists drafted questions about family dynamics, and discussed them with other primary-care physicians and clinical researchers. The final questionnaire asked about four problems or undesirable situations: disengagement (emotional distance), scapegoating (inappropriate blame), transfer of problems across generations (transfer of unnecessary burden from older to younger generations, trans-generationally displaced revenge), and undesirable behavior (co-dependence). Next, at six general-medicine clinics, doctors evaluated families that had a caregiver and a patient requiring long-term care. The results were analyzed by factor analysis. Cronbach’s α was computed, and criterion-related validation tests were done with three types of criteria: relationship before caregiving, ability to do activities of daily living (ADL), and the duration of care.

**Results:**

Results were obtained from 199 families. Among the caregivers, 79% were women and their mean age was 63 years. Among the patients, 71% were women and their mean age was 84 years. The results of factor analysis indicated that the scale was unidimensional. Cronbach’s α was 0.73. Not having a good relationship before caregiving was associated with significantly worse family dynamics scores, as was greater dependence regarding ADL.

**Conclusions:**

We developed a scale that enables physicians to assess the dynamics of families with a patient and a family caregiver. The scale’s scores are reliable and the results of validation testing were generally good. This scale holds promise as a tool both for research and for primary-care practice.

## Background

As of 2010, 23% of the people in Japan were over 65 years old, which was the highest percentage in the world [1]. The working-age population in Japan is 64% of the total, so there are 2.8 productive workers for each elderly person. This situation has serious social consequences, as the welfare and medical care of a large number of older people must be supported by a small number of working people [1]. Other developed countries have similar problems, but the situation in Japan is so severe that the international press has referred to it as the “Japan syndrome” [2].

In Japan, care for a frail elderly person is to a large extent shouldered by that person’s family. The burden on family members who provide that care is very large, and is an important problem [3]. In 2000, a publicly-funded insurance program was established to cover some of the costs of long-term care and thus to lessen the burden on family members. Through this program, older people can now receive nursing care services at home and in day-care centers, which reduces the amount of time that family members spend on such care.

Even when nursing care services are available, the burden on family members remains a problem [3]. Caregivers may be greatly burdened if the person for whom they are caring has trouble with activities of daily living (ADL) or has problems with cognitive functioning [4-8]. McDaniel et al. suggested that family dynamics are important in the care of frail elderly people [9], but no systematic methods have been established to measure family dynamics related to within-family care of elderly people. We developed a scale to quantify those family dynamics. Here we report on the development of that scale, and we present the results of reliability testing and validation testing.

## Methods

### The family dynamics scale

The family dynamics scale was developed by two primary-care specialists (TK and K Sato). They worked together with mentors who were specialists in clinical research and internal medicine (K Sakushima, S Fukuma, and S Fukuhara) and also with three other primary-care physicians (YM, SM, and TA). Overall, the aim was to identify problems or undesirable situations that might be alleviated via “intervention through a family system” [9] such as family consultations or family meetings. The developers identified four such problems or undesirable situations that might occur in caregiver-patient pairs (described below), and they wrote one question-item for each of those four problems. The content of the four question-items reflects both the clinical experience of the developers with regard to problems related to family dynamics, and concepts already discussed in this field [9]. The four are described below, with examples.

1. Disengagement. Example 1: a situation in which the caregiver feels no sense of fulfillment or accomplishment from caregiving, or feels no joy from the patient’s gratitude. Example 2: a situation in which the patient feels no gratitude to the caregiver. This is similar to “disengagement” as used by McDaniel et al. [9]. Disengagement was evaluated by observation of both the caregiver and the patient. For families in which the patient could not manifest a feeling of gratitude (for example, due to a neurologic condition), the evaluation was based on observation of the caregiver only.

2. Scapegoating (inappropriate blame). Example: “a situation in which the caregiver believes that the family would be happy if only the caregiving were easier”.

3. Transfer of problems across generations (specifically, transfer of unnecessary burden from older to younger generations, trans-generationally displaced revenge). Example: “a situation in which the patient compares the present with his or her past experiences as a caregiver, and, on that basis, is unnecessarily demanding of the caregiver”.

4. Undesirable behavior pattern. Example: “a situation in which the patient’s daily life has become very dependent on the caregiver, and can be sustained only by the caregiver, and in which the caregiver similarly feels dependent or reliant on the patient”. The latter may also be known as co-dependence, although that term was not used in the questionnaire.

The response options for those four question-items were as follows: definitely present (3 points); probably present (2 points); possibly present (1 point); and not present (0 points). Thus, lower scores indicate better family dynamics. For each patient-caregiver pair, the score was the mean number of points across the four items: lowest possible score = 0, highest possible score = 3. For example, a patient-caregiver pair that received 2 points on “disengagement”, 1 point on “scapegoating”, 2 points on “trans-generationally displaced revenge”, and 2 points on “undesirable behavior pattern” would have a score of 1.75, because 2 + 1 + 2 + 2 = 7, and 7/4 = 1.75. We call this scale the Index of Family Dynamics for Long-term Care (IF-Long).

### Participants and setting

Next, we conducted a multi-center observational cross-sectional study at six general-medicine clinics. Pairs of adult members of the same family, one of whom required long-term care and the other of whom gave that care (the patient and the caregiver, respectively) were eligible for inclusion if they regularly visited one of the six clinics. Each patient either was using Japan’s nursing-care (long-term) insurance program, had some limitation to their ability to visit the clinic, or had been given a diagnosis of dementia by consensus of multiple doctors at each of the six clinics.

### Procedures

Eligible families were asked to participate in the study when they came to the clinic for a regular outpatient visit. Those who consented were registered as participants and underwent a second examination. Caregivers and care managers of the patients received questionnaires by post. A physician who was certified as a primary care specialist responded to the four IF-Long questions. Here it is important to note that the IF-Long is intended to be used by a clinician, generally a primary-care physician, who has a long-standing relationship with the patient and the family.

Cognitive functioning of the patient was measured with the Functional Assessment Staging Test (FAST) [10]. The ability to do activities of daily living was quantified with the short version of the Functional Independence Measure (FIM) [11].

Caregivers were asked how many years they had been a caregiver for that patient, and how many hours per week they were devoting to being a caregiver. They were also asked about the quality of their relationship with the patient before caregiving began, i.e. whether that relationship was very good, good, not good, or bad. In addition, they were asked how well they understood the patient’s illness and medical condition, i.e. whether they understood it very well, to some extent, not very well, or not at all. For each caregiver, the following 9 items of information were also collected: (1) age, (2) sex, (3) kin relationship with the patient (spouse, child, etc.), (4) occupational-employment status, (5) whether or not they were raising a child, (6) whether or not they were raising a grandchild, (7) whether they visited the clinic regularly (i.e., about once per month or more frequently), (8) how they evaluated their own ability to tolerate stress (on a four-point scale, from very good to not good), (9) and how they evaluated their own economic situation (on a four-point scale, from “comfortable, not worrisome” to “difficult to live, very worrisome”).

Care managers were asked about the type of care services the patients were using: facility-based day care, facility-based day care that also included rehabilitation**,** overnight respite care, home-visit bath, home-visit nursing care, home rehabilitation, home attendant services, or some combination thereof.

### Data management and research ethics

A data center was established, and registered participants were assigned numbers. The questionnaires completed by the caregiver, care manager, and physician were sent directly to the data center by the person who completed them. Data were collected in a manner such that the data analyst could not identify individuals. The data center manager strictly protected the confidentiality of the participants. People who were directly involved in patient care were not allowed to view any of the raw data. The plan for this study was reviewed and approved by the research ethics committee of Kyoto University.

### Analyses

Means and standard deviations were calculated for the four IF-Long items. Factor analysis (Principal Factor Method) was done and Cronbach’s α was computed. The distribution of mean IF-Long scores was plotted.

It is reasonable to assume that if there are problems in family relationships before caregiving begins, then those problems become more apparent or more severe during caregiving, or as the health status of the patient worsens, or as the physical and psychological burdens on the caregiver increase. Thus, family dynamics during caregiving will be worse if the relationship before caregiving was not good. Therefore, as a validation test of the IF-Long, we hypothesized that IF-Long scores would be higher among those families in which the caregiver reported that the relationship before caregiving was not good. To test that hypothesis, we used one-way analysis of variance (ANOVA) and the Cuzick trend test [12] on the IF-Long scores and the caregiver’s response to the question “How was your relationship with the patient before you began caregiving?”

We also assume that greater functional dependence imposes greater physical and psychological burdens on the caregiver, and thus worsens family dynamics related to caregiving. Therefore, as another validation test of the IF-Long, we hypothesized that IF-Long scores would be higher among those families in which the patient had greater functional dependence (lower FIM scores). To test that hypothesis, we used one-way ANOVA and the Cuzick trend test [12] on the IF-Long scores and the FIM scores. For that purpose, the FIM scores were divided into three groups according to the mean ± 0.5 standard deviations (SD). The FIM score category was the independent variable and the IF-Long score was the dependent variable.

Finally, we assumed that family dynamics will be adversely affected if caregiving continues for a long time, and thus we hypothesized that families in which caregiving had lasted longer would have higher IF-Long scores. To test that hypothesis, the duration of care was divided into four categories (by quartiles), and differences in IF-Long scores among categories were tested by one-way ANOVA.

## Results

### Participants and factor analysis

**Figure 1:**
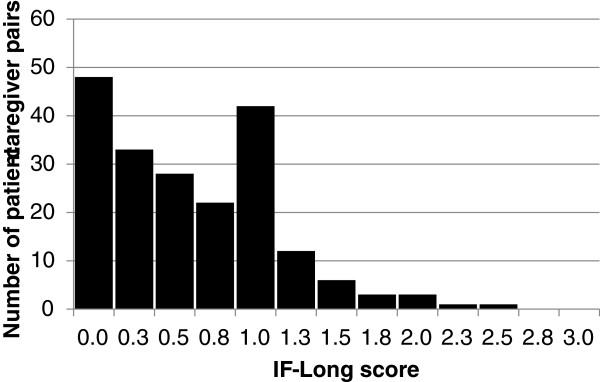
Distribution of IF-Long scores.

A total of 199 families were included. Among the patients, 71% were women and their mean age was 84 years. Among the caregivers, 79% were women and their mean age was 63 years. Personal attributes of the patients and caregivers are shown in Table 1. The mean duration of care was 5.2 years. The means of all four IF-Long items were less than 1.75 on the 0–3 scale (Table 2). From factor analysis, the four eigenvalues were 2.24, 0.76, 0.68, and 0.32, which indicates that the scale is unidimensional. Cronbach’s α was 0.73. The distribution of mean IF-Long scores is shown in Figure 1. The mean score was 0.61 with a standard deviation of 0.53.

**Table 1:** Characteristics of caregivers and patients

**Variables**		**n (%) or mean ± SD**
**Family**		
Number of family members	2	81 (40.7)
	3 or more	118 (59.3)
**Caregiver**		
Sex	Female	157 (78.9)
	Male	42 (21.1)
Age		63.2 ± 11.9
Kin relationship*	Spouse	53 (26.8)
	Child	76 (38.4)
	Daughter-in-law	29 (14.6)
	Son-in-law	1 (0.5)
	Sibling	5 (2.5)
	Grandchild	2 (1.0)
	Parent	32 (16.2)
Employed		67 (33.5)
Raising a child		21 (10.6)
Raising a grandchild		42 (21.1)
Assistant caregiver living together		86 (43.2)
Going to clinic regularly		127 (63.8)
Stress tolerance (self-reported)	Strong	19 (9.5)
	Somewhat strong	81 (40.7)
	Not very strong	74 (37.2)
	Not strong at all	25 (12.6)
Subjective economic condition	Comfortable, not worrisome	19 (9.5)
	Not very comfortable, but not worrisome	125 (62.8)
	Not comfortable, slightly worrisome	48 (24.1)
	Difficult to live, very worrisome	7 (3.5)
Years of caregiving		5.16 ± 4.73
Hours of caregiving per week		32.3 ± 39.1
Caregiver’s understanding of patient’s (medical) condition.	Understand it very well	78 (39.2)
	Understand it to some extent	115 (57.8)
	Don’t understand it well	6 (3.0)
	Don’t understand it at all	0 (0.0)
**Patient**		
Functional Independence Measure		33.9 ± 12.5
Functional Assessment Staging Test		3.59 ± 1.8
Services used	Home visit	75 (37.7)
	Day care	69 (34.7)
	All	117 (58.8)

*The total is 198 rather than 199, because of one missing datum.

**Table 2:** Mean, standard deviation, and factor loading of each IF-Long item

	**Mean**	**SD**	**Factor loading**
Emotional distance	1.73	0.77	.674
Inappropriate blame	1.66	0.67	.929
Transfer of problems across generations	1.41	0.61	.448
Undesirable behavior pattern	1.62	0.77	.525

### Criterion-related validation tests

#### Relationship before caregiving

**Figure 2:**
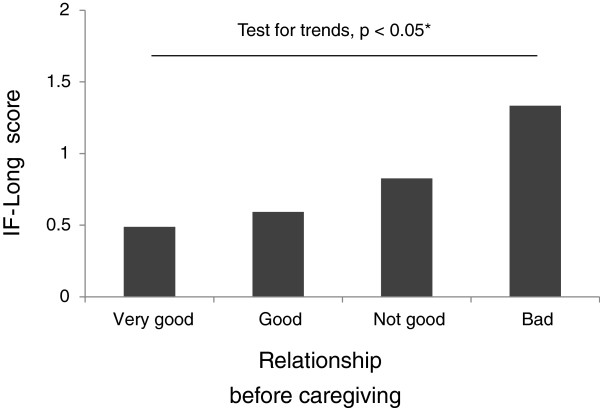
**Association of relationship before caregiving with IF-Long scores.** *Cuzick’s test for trends.

With the response to the question “How was your relationship with the patient before you began caregiving?” as the independent variable and the IF-Long score as the dependent variable (Figure 2), one-way ANOVA showed a main effect of the relationship before caregiving (F_3,192_ = 4.148, p < 0.01, Eta squared = 0.061). The Cuzick trend test revealed that not having a good relationship before caregiving was associated with higher IF-Long scores (Figure 2).

### FIM

**Figure 3:**
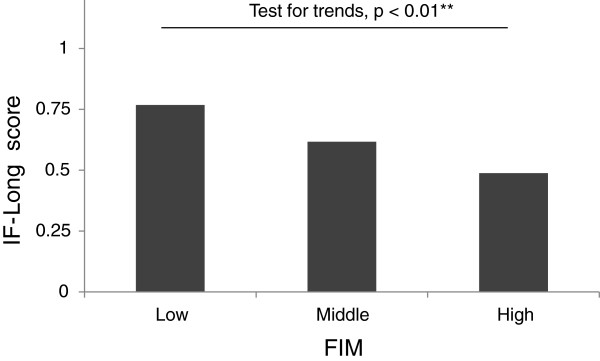
**Association of category of FIM score with IF-Long scores.** **Cuzick’s test for trends.

The mean FIM was 33.90 (SD = 12.51). The patients were categorized as follows: low FIM (FIM less than 27.65, that is, the mean minus 0.5 standard deviations), medium FIM (FIM of 27.65 or greater, but less than 40.15), and high FIM (FIM of 40.15 or greater, that is, the mean plus 0.5 standard deviations). One-way ANOVA with FIM category as the independent variable showed a main effect of FIM (F_2,196_ = 4.95, p < 0.01, Eta squared = 0.048). The Cuzick trend test revealed that being in a lower FIM category (i.e., having less functional independence) was associated with having a higher IF-Long score (Figure 3).

### Duration of care

**Figure 4:**
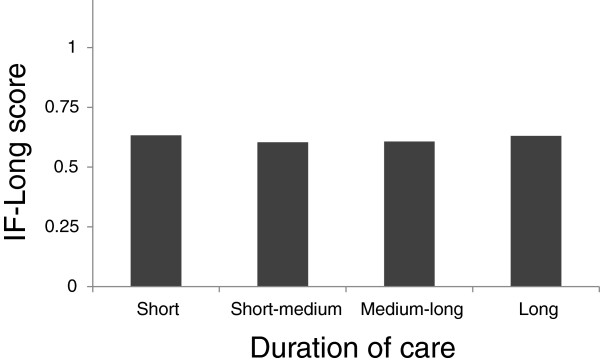
Association of category of duration of care with IF-Long scores.

The mean duration of care was 5.16 years (SD = 4. 73), with quartiles of 1.66, 4.21, and 6.33. The duration of care was categorized as follows: short (less than 1.66 years), short-to-medium (1.66 to 4.21 years), medium-to-long (4.21 to 6.33 years), and long (more than 6.33 years). One-way ANOVA with duration-of-care category as the independent variable did not show any significant differences (F_2,196_ = 0.039 , p = 0.99, Eta squared = 0.001, Figure 4).

## Discussion

The IF-Long is unidimensional, and its reliability (0.73) is sufficient, considering the small number of items. There was a small floor effect, but this does not adversely affect the clinical utility of the scale. A floor effect means that we cannot detect differences between families with good dynamics and families with very good dynamics. However, even if such differences could be detected, they generally would not affect clinical decisions. The small floor effect does not interfere with the important clinical task of detecting families with bad dynamics.

The FIM, which was assessed separately from the IF-Long, was associated with IF-Long scores in the hypothesized direction. That is, in general, IF-Long scores were high for families in which the patient’s FIM score was in a low category, and the IF-Long scores were low for families in which the patient’s FIM score was in a high category. The success of this validation test, that is, the inverse association of IF-Long scores with functional independence, indicates that the IF-Long score can be interpreted as an index of family dynamics.

In addition to the FIM, the quality of the relationship before caregiving, which was assessed separately from the IF-Long, was also associated with IF-Long scores in the hypothesized direction. That is, in general, the IF-Long scores were low for families in which the caregiver reported having had a good relationship with the patient before caregiving, and the scores were high for families in which the caregiver reported having had a bad relationship with the patient before caregiving. The success of this validation test indicates again that the IF-Long score can be interpreted as an index of family dynamics.

The category of the duration of care was not associated with IF-Long scores. This result is inconsistent with our hypothesis, and its explanation is unclear. One possibility is that the assumption on which the validation test was based is incorrect. That is, family dynamics might in fact not be associated with the duration of care. For example, family members might change their attitudes and behaviors over time, in a way that offsets the adverse effects of the duration of caregiving on family dynamics. This clearly shows the need for longitudinal studies of family dynamics.

One limitation of this study is the fact that we were unable to estimate test-retest reliability. Although test-retest reliability is important in this context, it may be quite difficult to measure. As noted by Nunnally and Bernstein [13], “the retest method often has serious problems, the most obvious being that memory for the first test usually influences the retest.” Therefore, if the test and retest are close together in time, then the former can bias the latter. Even if the test and retest are not close together in time, the test-retest method is still problematic because family relationships themselves may change in the interval. As noted by DeVellis [14], measured values of test-retest reliability give information about a scale “only when we are highly confident that the phenomenon has remained stable. Such confidence is not often warranted”. If a separate, independent indictor of family dynamics were available, and if it were practical to re-evaluate patient-caregiver pairs over intervals that are neither too short nor too long, then one might be able to estimate test-retest reliability.

Another limitation is that the IF-Long was tested only with families in which the patients were seen in a clinic run by a primary-care physician. Nonetheless, some regional diversity was achieved by recruiting patients from six different medical clinics in areas ranging from rural to urban. Reliability coefficients above 0.7 are often considered to be sufficient for some purposes, but there were only four items, which may not be entirely adequate for a psychological scale, so one direction for future work might be to slightly increase the number of items. However, the fact that the scale is short should be an advantage in busy outpatient settings.

The four IF-Long items were developed through literature research and discussion among primary-care specialists who were well-versed in family-oriented primary care. Nonetheless, it is clear that the validation tests reported here are not conclusive or complete. Further validation testing with qualitative studies involving other stakeholders would be helpful to overcome this limitation. Specifically, information regarding family-dynamics constructs could come from focus-group discussions with patients and caregivers in future studies. In addition, because the IF-Long was developed to measure family dynamics between givers and receivers of long-term care, it might not be applicable to family dynamics related to other health issues.

One advantage of the IF-Long is that it has only four items, so it should be easy to use even during busy primary-care consultations. One disadvantage is that it can be used only by a trained primary-care physician who is the primary doctor of the family concerned and who has already established a healthcare relationship with that family. We hope to write a manual and develop a training program to help physicians use the IF-Long.

Scales such as the Family Adaptability and Cohesion Evaluation Scale III [15] have been used in family studies and family therapy, but to the best of our knowledge the IF-Long is the first scale developed for primary-care physicians who want to measure family dynamics as they relate to long-term care. Considering the IF-Long as a tool for future research, we note that information about how family dynamics are related to the family’s living arrangements, the family’s economic situation, and kin relationships could be important, and the IF-Long may be useful in studies of those associations. In addition, while the IF-Long was developed as a four-item scale, interesting aspects of family dynamics might be revealed by the responses to each of the items individually, particularly if there are associations with, as mentioned above, living arrangements, economic situation, and kin relationships.

The IF-Long may also be useful in studies of the effects of family dynamics on many aspects of long-term care, such as, for example, the relationship between family dynamics and the burden on caregivers. If a physician intervenes to maintain or improve the functioning of the family system, the IF-Long might be used as one of the outcome measures, and thereby it could help physicians provide more individualized care. In Japan, primary-care physicians cannot easily refer families to a family therapist, so primary-care physicians themselves must take on that role. Physicians who assume those responsibilities may find the IF-Long to be useful. They might also need methods to comprehensively measure and evaluate family dynamics that are not limited to long-term care relationships, and methods to measure family dynamics that can be applied to other health issues.

## Conclusions

The IF-Long can be used in primary care to reliably assess family dynamics related to long-term care. While the scale might be improved by inclusion of more items, and further validation studies could be useful, the IF-Long holds promise as a tool both for research and for primary-care practice.

## Competing interests

All authors declare that they have no competing interests.

## Authors’ contributions

All authors made substantial contributions to conception and design of the study, and to the interpretation of data; TW was involved in psychometric analyses; TK, KS, SF, and SF were involved in drafting the manuscript and revising it critically for important intellectual content; and KS, YM, SM, and TA were involved in conducting the research. All authors read and approved the final manuscript.

## Pre-publication history

The pre-publication history for this paper can be accessed here:

http://www.biomedcentral.com/1471-2296/15/134/prepub

## References

[B1] OECD Country Statistical Profile 2013 Japan: OECD Publishing [http://www.oecd-ilibrary.org/economics/country-statistical-profile-japan_20752288-table-jpn]

[B2] The Economist The future of Japan: The Japan syndrome 2010 [http://www.economist.com/node/17522568] (Accessed on 2013.06.30)

[B3] Tamiya N Noguchi H Nishi A Reich MR Ikegami N Hashimoto H Shibuya K Kawachi I Campbell JC Population ageing and wellbeing: lessons from Japan’s long-term care insurance policy Lancet 2011 378 1183 1192 10.1016/S0140-6736(11)61176-8 21885099

[B4] Hiramatu M Kondou K Umehara K Kuze J Higuchi K Kazokukaigosya no futankan to kanren suru inshi no kenkyu (Analysis of factors associated with burden on family caregivers) J Health Welfare Stat 2006 53 19 24

[B5] Ohyama N Suzuki M Yamada K Kazokukaigosya no shukantekikaigofutan ni okeru kanren youin no bunseki (Analysis of factors associated with the subjective burden on family caregivers) Rounenkangogaku 2001 6 58 66

[B6] Wakui T Saito T Kai I Zaitakukaigosya no syakai and yoka katsudou ga kaigofutankan ni ataeru eikyo (Impacts of social and leisure activities on the burden on family caregivers) Res Grant Proceed Yasuda Mental Health Foundation 2006 42 210 218

[B7] Kishida K Tanigaki S Zaitaku sa-bisu nani ga tarinainoka kazokukaigosya no kaigofutan no bunseki (What is lacking in home care service - Analysis of the burden on family caregivers) Jap J Health Econ Policy 2007 19 21 35

[B8] Makizako H Abe T Abe K Kobayashi S Koguchi R Onuma T Shimada H Nakamura Y Factors burdening the caregiving relatives of community-dwelling disable Japanese people Nihon Ronen Igakkai Zasshi 2008 45 59 67 10.3143/geriatrics.45.59 18332574

[B9] McDaniel SH Shore B McDaniel SH, Campbell TL, Hepworth J, Lorenz A Anticipating loss: healthcare for older patients and their family caregivers Family-Oriented Primary Care 2005 2 New York: Springer 242 260

[B10] Reisberg B Ferris SH Anand R De Leon MJ Schneck MK Buttinger C Borenstein J Functional staging of dementia of the Alzheimer type Ann N Y Acad Sci 1984 435 481 483 10.1111/j.1749-6632.1984.tb13859.x

[B11] Yamada S Liu M Hase K Tanaka N Fujiwara T Tsuji T Ushiba J Development of a short version of the motor FIM for use in long-term care settings J Rehabil Med 2006 38 50 56 10.1080/16501970510044034 16548088

[B12] Cuzick J A Wilcoxon-type test for trend Stat Med 1985 4 87 90 10.1002/sim.4780040112 3992076

[B13] Nunnally JC Bernstein IH Psychometric Theory 1994 3 New York: McGraw-Hill 254

[B14] DeVellis RF Scale Development 2012 3 Los Angeles: Sage 52

[B15] Olson DHL Family inventories: Inventories used in a national survey of families across the family life cycle 1992 University of Minnesota: Family Social Science

